# Annotations

**Published:** 1889-04-06

**Authors:** 


### Annotations.

**The Children of Inhumanity.** It is, perhaps, quite impossible for moderately prosperous people to realise what the world is like to certain classes of children in London and other large towns. The cold indifference of the men and women they see in the streets; the hard and relentless cruelty which reigns unchecked in the dens they know as homes; the cheerless day and the hopeless night—these must produce in many children, either a deadened and callous indifference, or a feeling of unremitting misery and utter despair. The power of our countrymen is irresistible when they combine for self-defence or for self-advancement; but it dwindles wofully when the object is that of helping others, and especially if those others be of no account in the financial, the social, or the political sphere. Pure and energetic benevolence is not a relatively large quantity in the average human being. We do not wish for a moment to decry the many good works done by a few of our compatriots; they are beyond all praise. But what of the many—the many who, if they would unite, could remove whole mountains of misery and suffering that weigh down the helpless as with a world-nightmare? They do not care; or if they do care, their care is not anxious enough to bring about the raising of a single finger to help. The Society for the Prevention of Cruelty to Children has been at work for several years; the few persons who compose it have striven nobly, and what has been accomplished up to the present time has been almost entirely done by them. But should not all civilisation itself be one vast society for the prevention of cruelty to children, as well as for the prevention or extinction of all other abominations and iniquities? A stage of progress has at length been reached. A Bill is printed and "backed." Members of Parliament of all parties have united in the backing of it. Among these are Sir Stafford Northcote, Mr. John Morley, Sir Henry James, Sir Robert Fowler, Sir Albert Rollit, and others. That is well; but it needs to be got through the House, and when it has passed the House it will still need to be universally enforced. It can neither pass the Legislature nor be of service after it is passed if the public do not give it their active and urgent sympathy. Are we waiting for something to arouse that sympathy? Let anyone who feels indifferent to the woes of the poor and humanity-forsaken children of large towns write to Mr. Benjamin Waugh, the hon. secretary to the Society for the Prevention of Cruelty to Children, for two or three of the society's latest annual reports. Nothing more is needed to convert every man and woman of the least kindness of heart into an active and steady supporter both of the Bill and of the society. Benevolence that does not fly to its object is either sick or dead, and needs to be cured or buried.

**Leisure and Temper.** "Leisure" is a little out of date in large towns. In the country most men have more than they know what to do with. Some people cannot enjoy leisure at all. It seems as if Nature had left them minus a special sense. Others are never so happy as when mooning and dreaming by the sea, in the country lanes, or in the restful seclusion of the library. Leisure, like sleep, is a great restorer. It "knits the ravelled sleeve" of many a care. The human brain, like the human skin, cannot stand more than a certain amount of friction. If one part of the skin be persistently rubbed, it becomes at length so sensitive that even the soft touch of a child will give it pain. Let the town man with his "paper skin" try haymaking, or cricket, or other such unaccustomed exercise, and a painful redness, or even a blister or two on the palm will soon convince him that too much friction is bad. It is exactly so with the brain. The man who has no daily period of genuine leisure cannot help his brain becoming as sensitive as the reddened or blistered palm of the unaccustomed haymaker. When things have reached that pass, the temper is apt to become wonderfully irritable. The smallest contradiction will produce an explosion. "Anger," says the Latin primer, "is brief madness." The man whose want of leisure has brought his brain into a condition of extreme hypersensitiveness has sometimes a dozen attacks of such brief madness in a day. Each attack, with its resulting temporary exhaustion and disordered mental vision, can hardly last altogether less than half an hour. The hardworking but leisureless man may therefore find himself after a few years' strain deprived of his best, because his calmest, judgment for five or six hours a day. If, under these circumstances, he often arrives at foolish decisions, and sometimes even brings himself into difficulties of the most critical and dangerous kind, who will be surprised at it? Sleep everyone knows to be absolutely indispensable. A certain amount of waking leisure is quite as essential to perfect health. The natural periods of the day for such repose of mind are the meal-times and the evenings. Every meal—and most town people require three or four a day—should be a time of physical satisfaction and mental repose. At the end of the day's work there should be two or three hours of entire freedom from brain stress, with simple mental pleasures. A man who will fulfil these conditions may not only hope to live his "full span" of life, but also to give the world the best that is in him, and to get out of it the best that it has to offer.

**The Burden of the Books.** According to Messrs. Low and Sons' "Annual Supplement" there has been a large increase in the number of published books during the past year. This literature is divided into thirteen classes. Among these no fewer than 912 separate works are devoted to theology, 172 to law, and 199 to medicine. That is terrible! Who buys all these books it is difficult to say; who reads them it is impossible to even conjecture. The students of theology and of law may, if they choose, speculate on the destiny of the thousand odd publications which fall to their share. We are more immediately concerned with the 199 separate literary works that have appeared like stars or meteors in the already crowded sky of medicine. What in the world can they all be about, and who in the world has written them? It is well within our knowledge that the medical profession in this country is absolutely blinded and dazed with the infinite variety of new works published annually on every conceivable subject. Is it possible that there are in Great Britain so many medical men of exceptional experience, practical skill, and literary power as to produce about 200 separate works every year? Supposing each author to produce only one volume in ten years, we should have among the 30,000 medical men of the country no fewer than 2,000 original authors—that is, one doctor in every fifteen, according to this calculation, is an author. What is the explanation? Who are these burning and shining lights? Are they the older or the younger men? Is it experience, or is it ambition that so madly rushes into print? We will hazard the conjecture that of the supposed 2,000 medical authors not 200 are over thirty-five years of age. But of medical men under that age, not one in a hundred ought to presume to approach his fellows in what used to be the formal and dignified act of writing a book. The truth is that many men who are really capable of writing, and who have something to say, will not put pen to paper lest they should find themselves taken for advertising bookmakers. The critical faculty is not strongly developed in doctors, and in the crowd of claimants for their suffrages the man of real merit is very apt to be overlooked. There is probably no way of checking the ever-increasing deluge of medical literature; but it would be a good rule to regard every medical man under forty who publishes a book or a pamphlet, with suspicion, and to have a black—or at least a dark brown—list to which the names of all such persons could be temporarily relegated until they had by their honest practical work purged themselves of the doubtful morality of roundabout advertising. Failing this, we can but protest, as we do with all possible energy, against the overwhelming flood of printer's ink which, being charged with none of the antiseptic properties of brains or experience, threatens to swamp and drown the judgment of the profession without hope of remedy.

